# Correction: Large-area thin-film synthesis of photoactive Cu_3_PS_4_ thiophosphate semiconductor with 0–14 pH stability range

**DOI:** 10.1039/d6sc90013e

**Published:** 2026-01-30

**Authors:** Lena A. Mittmann, Javier Sanz Rodrigo, Eugène Bertin, Giulia Dalmonte, Jean-Claude Grivel, Ivano E. Castelli, Andrea Crovetto

**Affiliations:** a National Centre for Nano Fabrication and Characterization (DTU Nanolab), Technical University of Denmark 2800 Kongens Lyngby Denmark mittma@dtu.dk ancro@dtu.dk; b Department of Energy Conversion and Storage (DTU Energy), Technical University of Denmark 2800 Kongens Lyngby Denmark

## Abstract

Correction for ‘Large-area thin-film synthesis of photoactive Cu_3_PS_4_ thiophosphate semiconductor with 0–14 pH stability range’ by Lena A. Mittmann *et al.*, *Chem. Sci.*, 2025, **16**, 21862–21873, https://doi.org/10.1039/D5SC05882A.

The original version of this article contained typographical errors in the following section of text:

At the CBM, the dominant antibonding interactions are between S 3p states and P 4s states, with a higher P 4s character at the Γ point than at the CBM on the Γ–X path. Again, the situation is analogous to S 3p/In 6s mixing at the CBM of chalcopyrites and S 3p/Sn 6s mixing in kesterites, pointing to the similar role of P, In, and Sn across these copper-based sulfides, and to phosphorus behaving much like a post-transition metal cation.

Here, P 4s should be P 3s, In 6s should be In 5s and Sn 6s should be Sn 5s. This typographical error does not affect any of the conclusions drawn within the manuscript.

The Royal Society of Chemistry apologises for these errors and any consequent inconvenience to authors and readers.

